# Mixture Theory-Based Finite Element Approach for Analyzing the Edematous Condition of Biological Soft Tissues

**DOI:** 10.3390/bioengineering11070702

**Published:** 2024-07-11

**Authors:** Satoko Hirabayashi, Masami Iwamoto, Xian Chen

**Affiliations:** 1 Human Science Research Domain, Toyota Central R&D Labs., Inc., Nagakute, Aichi 480-1192, Japan; iwamoto@mosk.tytlabs.co.jp; 2 Graduate School of Sciences and Technology for Innovation, Yamaguchi University, Ube, Yamaguchi 755-8611, Japan; xchen@yamaguchi-u.ac.jp

**Keywords:** finite element method, pore water pressure, mixture theory, membrane, hydrated poroelastic material, pitting edema, edematous condition

## Abstract

In hydrated soft biological tissues experiencing edema, which is typically associated with various disorders, excessive fluid accumulates and is encapsulated by impermeable membranes. In certain cases of edema, an indentation induced by pressure persists even after the load is removed. The depth and duration of this indentation are used to assess the treatment response. This study presents a mixture theory-based approach to analyzing the edematous condition. The finite element analysis formulation was grounded in mixture theory, with the solid displacement, pore water pressure, and fluid relative velocity as the unknown variables. To ensure tangential fluid flow at the surface of tissues with complex shapes, we transformed the coordinates of the fluid velocity vector at each time step and node, allowing for the incorporation of the transmembrane component of fluid flow as a Dirichlet boundary condition. Using this proposed method, we successfully replicated the distinct behavior of pitting edema, which is characterized by a prolonged recovery time from indentation. Consequently, the proposed method offers valuable insights into the finite element analysis of the edematous condition in biological tissues.

## 1. Introduction

Hydrated soft tissue involves complex interactions between solids and fluids. Fluids such as blood, intracellular fluid, extracellular fluid, and lymph fill numerous fine voids within the solid skeleton, thereby hydrating the tissue. The membrane surrounding the tissue regulates the transmembrane fluid flow and determines the volume and pressure of fluids contained within the tissue. An accumulation of fluid in the interstitial space, known as edema, occurs when capillary filtration exceeds the capacity of lymphatic drainage, leading to noticeable clinical signs and symptoms. In some cases of edema, specifically pitting edema, an indentation that is induced by pressing a finger on the tissue persists even after the finger is removed. Pitting edema is associated with decreased plasma oncotic pressure and disorders caused by increased capillary pressure, such as deep vein thrombosis, congestive heart failure, and iliac vein compression. The depth and duration of the pitting are used to determine the treatment response [1,2,3,4].

The deformation of hydrated soft tissue is a multi-physics and multi-scale problem involving both fluids and solids. While fluid–solid interactions occur at a microscopic level along intricate fluid–solid boundaries, fluid movement and solid deformation stress are macroscopic phenomena. These complex phenomena can be investigated by replication and observation via finite element method (FEM) simulations. However, traditional FEM simulations require an unrealistically fine finite element mesh to replicate the fluid–solid boundaries in hydrated soft tissues accurately. We previously considered averaged fluid–solid interactions based on mixture theory, adopting a macroscopic approach to model the tissues, and performed finite element analyses of hydrated soft tissue behavior without using fine meshes [5]. Using a similar method, Klahr et al. [6] demonstrated that a constant tissue volume can be obtained by conducting a displacement-controlled monotonic uniaxial test on undrained hydrated tissues. However, to the best of our knowledge, the edematous condition has not been analyzed in terms of the increase in tissue volume resulting from excess fluid accumulation, solid deformation stress, and fluid pressure. Therefore, the objective of this study was to develop a novel FEM for effective edema analysis.

Two different numerical approaches are generally used for a coupled dynamic analysis of flow and deformation in porous materials: methods that consider pressure and solid displacement as unknowns (u-p formulation) [7,8,9,10], and methods that include pressure, solid displacement, and fluid velocity vectors as unknowns (u-w-p formulation) [11,12,13,14]. These methods are widely applied, ranging from soil analysis [15] to the study of organs such as lungs [16] and intervertebral discs [17]. In our previous study, pressure and solid displacement served as the nodal unknowns (u-p formulation) [5]. However, significant numerical errors arose in the edema analyses; for instance, the total volume changed despite the tissue being surrounded by a completely impermeable membrane. In this study, we included the fluid velocity vector in the nodal unknowns (u-w-p formulation) based on studies that have reported reliable results [13,17,18,19,20]. During the three-dimensional (3D) FEM analyses performed in these studies, the fluid velocity vector at each node was represented by a set of three independent scalar nodal unknowns based on a set of base vectors. The transmembrane flow must be one of the three scalar nodal unknowns to maintain it as a Dirichlet boundary condition without constraining the flow parallel to the membrane. This implies that one of the bases should align with the direction of the transmembrane flow, which is perpendicular to the membrane. However, the complex shape and deformation of the tissue make this challenging. To address this, we introduced a local coordinate system at each time step and node, linking the variables in the local and global coordinate systems via coordinate transformation. Using these methods, we reproduced a pitting edema indentation test and analyzed the depth and duration of the pitting to evaluate the proposed method.

## 2. Methods

### 2.1. Theoretical Formulations

The unknown variables considered in this study were the solid displacement, interstitial fluid pressure, and fluid relative velocity, based on previous studies that reported reliable results [13,17,18,19,20]. The governing equations and their derivations are detailed in Appendix A.

Using the Galerkin method, we discretized the set of governing equations with hexahedral elements, employing continuous bilinear interpolations for the solid and fluid variables and discontinuous constant pressures, as indicated in Equations (A7), (A15), and (A21). 

The vectors and tensors were represented using a set of base vectors, with their coefficients arranged as rows and combined into a matrix. The same set of base vectors, $e_{1}$, $e_{2}$, and $e_{3}$, was used throughout the analysis. A new set of base vectors, ${\tilde{e}}_{1}$, ${\tilde{e}}_{2}$, and ${\tilde{e}}_{3}$, and two sets of three scalars (the default notation $\left\{W\right\}\equiv {\left\{\begin{array}{ccc} W_{1} & W_{2} & W_{3} \end{array}\right\}}^{T}$ and a new notation $\left\{\tilde{W}\right\}\equiv {\left\{\begin{array}{ccc} {\tilde{W}}_{1} & {\tilde{W}}_{2} & {\tilde{W}}_{3} \end{array}\right\}}^{T}$) were used to represent the fluid relative velocity vector W as

(1) $$W=W_{1}e_{1}+W_{2}e_{2}+W_{3}e_{3}={\tilde{W}}_{1}{\tilde{e}}_{1}+{\tilde{W}}_{2}{\tilde{e}}_{2}+{\tilde{W}}_{3}{\tilde{e}}_{3}.$$

The new set of base vectors could vary for each node and time step.

**Figure 1:**
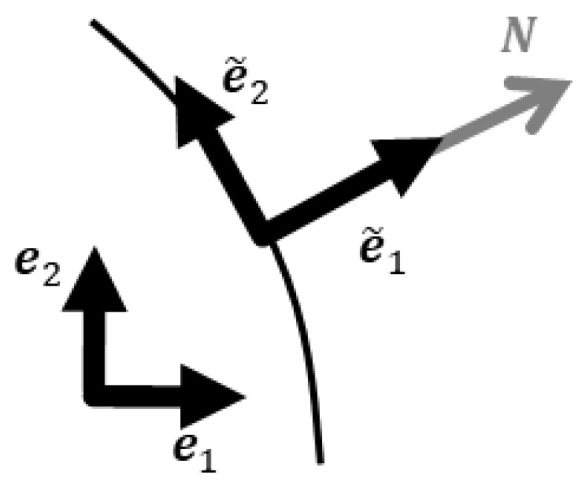
Coordinate transformation.

After rewriting the governing equations as a matrix using the default notation, we substituted the following relationship:

(2) $$\left\{W\right\}=\left[P\right]\left\{\tilde{W}\right\},$$

where

(3) $$\left[P\right]\equiv \left[\begin{array}{ccc} e_{1}\cdot {\tilde{e}}_{1} & e_{1}\cdot {\tilde{e}}_{2} & e_{1}\cdot {\tilde{e}}_{3}\\ e_{2}\cdot {\tilde{e}}_{1} & \ddots & \vdots \\ e_{3}\cdot {\tilde{e}}_{1} & \cdots & e_{3}\cdot {\tilde{e}}_{3} \end{array}\right]$$

denotes the coordinate transformation matrix at each node. The new notation $\left\{\tilde{W}\right\}$ became the nodal unknowns. Among these new base vectors, one was specifically oriented perpendicularly to the membrane at its nodes. This particular orientation was crucial for representing the transmembrane flow as the corresponding component of the new notation $\left\{\tilde{W}\right\}$ and establishing the Dirichlet boundary condition. Figure 1 depicts the new set of base vectors at a membrane node.

**Figure 2:**
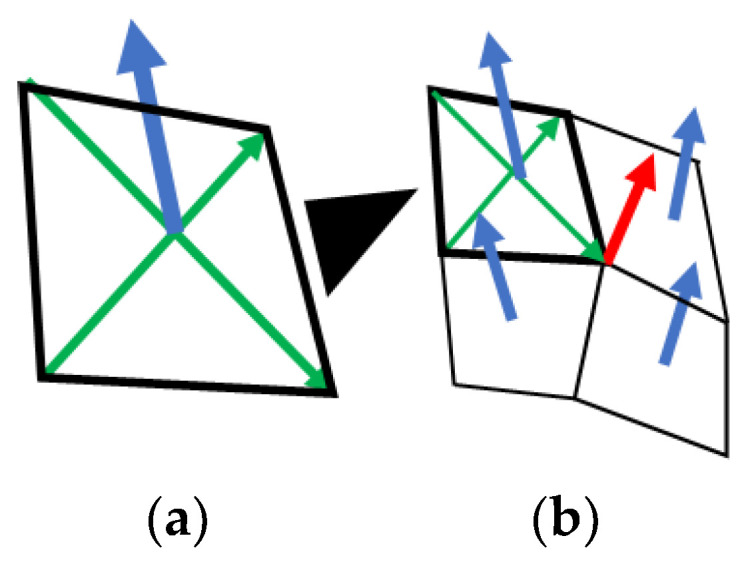
Unit vector normal to membrane. (**a**) The normal unit vector of each surface element was obtained by calculating the cross product of the two diagonal vectors. (**b**) The normal unit vector at each node was defined as the unit vector parallel to the sum of the normal unit vectors of all surface elements that shared the node under consideration.

The unit vector normal to the membrane at each node (N in Figure 1) was calculated as follows. Initially, the membrane was divided into surface elements corresponding to the faces of the hexahedral elements defining the volume domain. For each surface element, the normal unit vector (blue arrow in Figure 2a) was obtained by calculating the cross product of the two diagonal vectors (green arrows in Figure 2a). Subsequently, the normal unit vector at each node on the membrane (red arrow in Figure 2b) was defined as the unit vector parallel to the sum of the normal unit vectors of all surface elements that shared the node under consideration (blue arrows in Figure 2b).

We modeled the solid skeleton (drained solid constituent) as a homogeneous hyperelastic material defined by the St. Venant–Kirchhoff constitutive model with elastic potential ψ, as follows:

(4) $$\psi =\frac{\lambda }{2}{\left(trE\right)}^{2}+\mu tr\left(E^{2}\right),$$

where λ and μ represent the Lamé’s constants and E denotes the Lagrangian strain. Using the coefficients of $S^{E}$ and E in matrix notation, the second Piola–Kirchhoff stress $S^{E}$ was represented as

(5) $$S_{ij}^{E}=\frac{\partial \psi }{\partial E_{ij}}.$$

Substituting Equation (4) into Equation (5) yields

(6) $$S^{E}=\lambda \left(trE\right)I+2\mu E.$$

The Poisson’s ratio and Young’s modulus of the skeleton were set to 0 and 30.0 kPa, respectively, implying that λ = 0.0 and μ = 15.0 kPa. In addition, the Darcy permeability tensor of the mixture was assumed to be κ=κI**,** with κ = $5.0\times 10^{-5}$ m^4^/N-s. The initial solid volume fraction was assumed to be 0.5.

We used the Gauss integration scheme for spatial integration and the backward Euler algorithm for time linearization. The resulting set of equations was solved iteratively, using the Newton–Raphson procedure.

### 2.2. Numerical Verification

**Figure 3:**
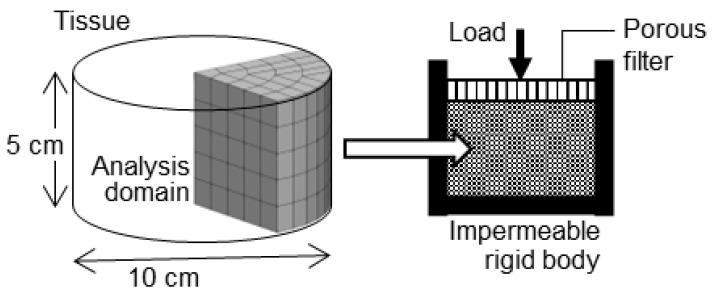
Geometry and mesh of analytical model for verification.

For verification, we prepared a cylindrical analytical model with a radius of 5 cm and a length of 5 cm, featuring no deformation stress and no pore water pressure. The lateral and bottom surfaces of the model were surrounded by an impermeable rigid body (Figure 3). Subsequently, we performed creep and stress relaxation analyses, applying a load to the permeable filter at the top surface. During the creep analysis, a step function stress was applied:

(7) $$\sigma =\left\{\begin{array}{ccc} \sigma_{0} & ; & t>0\\ 0 & ; & t=0 \end{array}\right.,$$

where $\sigma_{0}$ = 1.0 kPa. During stress relaxation, the forced displacement was defined by the following ramp function:

(8) $$d=\left\{\begin{array}{ccc} v_{0}t & ; & 0\leq t<t_{0}\\ v_{0}t_{0} & ; & t>t_{0} \end{array}\right.,$$

where $v_{0}$ = 2 m/s and $t_{0}$ = 1 ms. Leveraging symmetry constraints, only one fourth of the total analysis domain was analyzed.

### 2.3. Reproduction of Indentation Test under Pitting Edema

**Figure 4:**
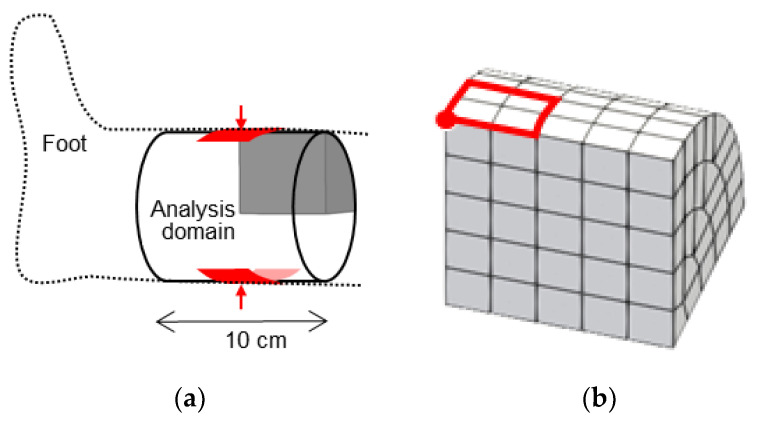
(**a**) Geometry of the analytical model for the indentation test. (**b**) Mesh of the analytical model. The red square and point indicate the loaded surface and the point at which solid displacement were observed, respectively.

The confined problem solved in the previous section is essentially a 1D problem involving only axial flow without circumferential flow. Thus, it does not validate the new method but merely verifies the formulation and implementation through viscoelastic behavior. To verify the effectiveness of the developed method, we performed 3D analyses by modeling indentation tests under two states: Normal and Edema. To model a part of a lower leg, we prepared a cylindrical analytical model with a radius of 5 cm and a length of 10 cm, featuring no deformation stress and no pore water pressure (Figure 4); this state is referred to as the “Normal state”. The other state, called the “Edema state”, was prepared by inflating the Normal state up to 130% using extra fluid (Table 1). After reaching a steady state, the pore pressure in the edema becomes uniform. The initial solid and fluid volume fractions were uniform in each state. In both the states, we restricted the axial length to 10 cm, and the transmembrane flow was set to zero across the entire surface, representing its encapsulation by impermeable membranes. As the external pressure was set to zero, the pore water pressure in the Edema state was increased to balance it with the solid deformation stress.

We performed an indentation analysis in both the Normal and Edema states by applying an inward load to the lateral surface of the cylinder, modeling the situation of pinching a part of the thigh between two fingers (Figure 4). We applied different loads for each state, to unify the depth of the indentations under both states (Table 1). After inducing an indent, we removed the load and continued the analysis. By leveraging symmetry constraints, only one eighth of the total analysis domain was investigated.

## 3. Results

### 3.1. Numerical Verification

**Figure 5:**
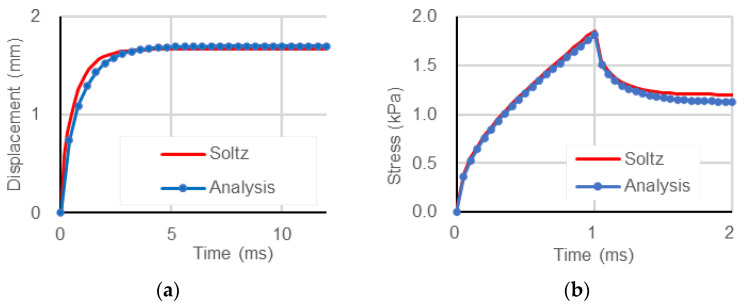
(**a**) Creep. (**b**) Stress relaxation. The red and blue lines indicate the theoretical solutions derived by Soltz and Ateshian [21] and the results obtained by our analysis, respectively.

Figure 5a shows the evolution of the axial displacement of the top surface during the creep test, while Figure 5b shows the evolution of the axial stress on the top surface during the stress relaxation test. Both figures also display the theoretical solutions derived by Soltz and Ateshian [21], which closely match the analysis results.

### 3.2. Indentation Test under Pitting Edema

**Figure 6:**
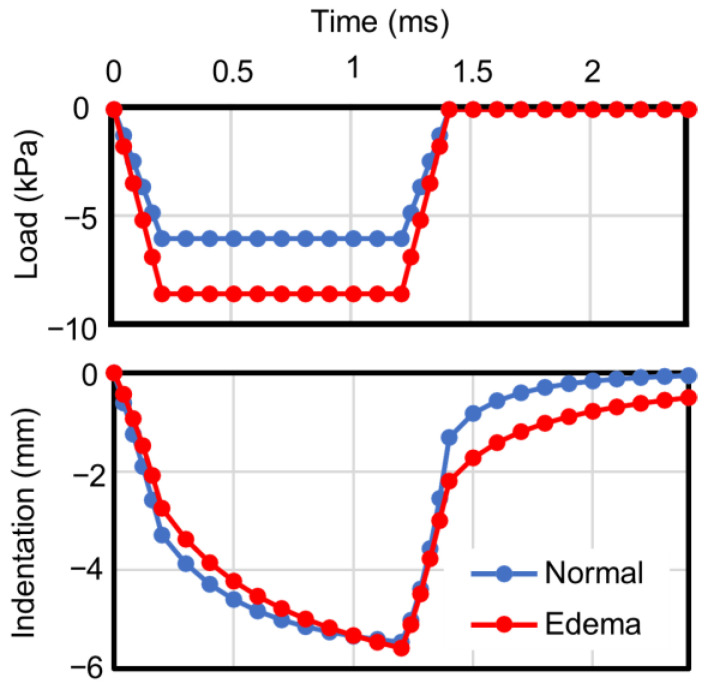
Applied forces and indentation depth in Normal and Edema states.

Figure 6 shows the evolution of the applied forces and indentation depth during the indentation tests. The indentation depth was measured at the red point in Figure 4b. In both the Normal and Edema states, deformations continued even after the loads became constant, indicating that the mixed material exhibited viscoelastic properties due to the interaction between the interstitial fluid and solid skeleton. In the Edema state, a larger load was required to achieve the same indentation depth as that in the Normal state. Furthermore, the indentation in the Edema state persisted for longer than that in the Normal state. As we achieved control of the fluid flow through deformable soft membranes without impeding other components, the total tissue volumes remained constant throughout the tests, with the fluid being able to flow along the membrane.

**Figure 7:**
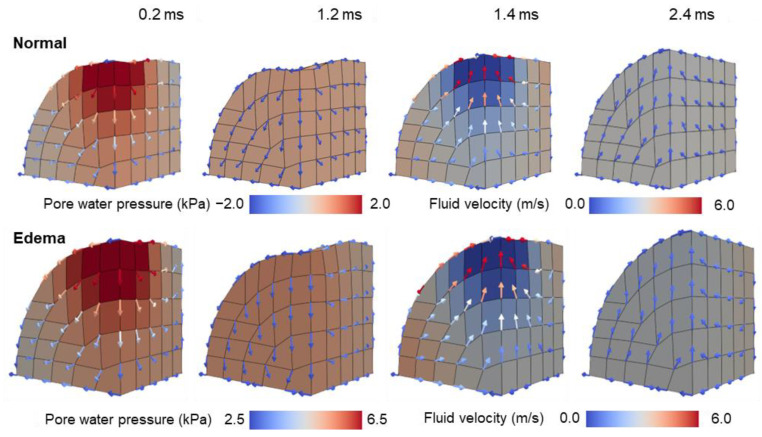
Distribution of the pore water pressure with fluid flow vector plots during the indentation test in the Normal and Edema states (1/8 symmetry).

Figure 7 depicts the distribution of the pore water pressure, with the arrows indicating the fluid relative velocity w (Equation (A10)). Owing to higher pore water pressure in the Edema state than in the Normal state, the color contour range was adjusted accordingly. In the loaded area, the pore water pressure increased as the load increased (time: 0–0.2 ms), dissipated when the load remained constant (time: 0.2–1.2 ms), decreased as the load decreased (time: 1.2–1.4 ms), and dissipated when the load remained constant (time: 1.4–2.4 ms).

**Figure 8:**
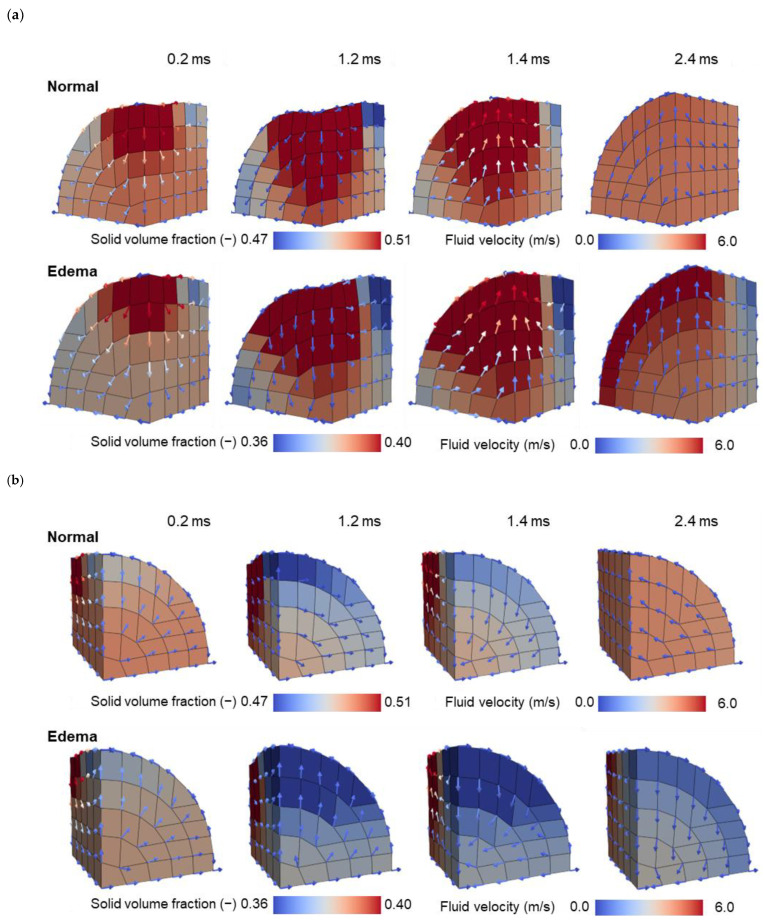
Distribution of the solid volume fraction with fluid flow vector plots during the indentation test in the Normal and Edema states (1/8 symmetry). (**a**) View from center. (**b**) View from edge.

We can calculate the solid volume fraction from the volume change of the mixture (Appendix B). Figure 8 presents the distribution of the solid volume fraction calculated using Equation (A22), with arrows indicating the fluid relative velocity w. As the solid volume fraction was higher in the Edema state than in the Normal state, the color contour range was adjusted accordingly. Notably, the solid fraction increased in the compressed area due to fluid outflow. Conversely, the solid fraction decreased in other areas, conserving the total volume of the mixture.

As shown in Figure 7 and Figure 8, fluid flowed according to the gradient of the water pressure. It was expelled from the point where the load was applied, changed direction upon encountering opposite-flowing fluid at the symmetry plane, and gradually diminished as it extended, eventually halting at the top or bottom of the cylinder. Owing to the control of the fluid flow through deformable soft membranes, the fluid flowed along the membrane without leaking out. Under a constant load over time, a difference in the fluid flow between the Edema and Normal states was observed at locations distal to the load application point (time: 1.2 ms). After unloading, both the solid and fluid moved to return the mixture to its original state in both the Normal and Edema states. When the Normal state had almost returned to its original condition, the Edema state had not yet reverted to its baseline (time: 2.4 ms).

## 4. Discussion

### 4.1. New Approach for Simulating Edematous Condition of Soft Biological Tissues

We have proposed a mixture theory-based approach for simulating the edematous condition of soft biological tissues, characterized by an excessive accumulation of fluid within hydrated tissue encapsulated by impermeable membranes. Our analysis utilized the solid displacement, interstitial fluid pressure, and fluid relative velocity as the unknown variables. By implementing coordinate transformation at each time step and node, we successfully incorporated the transmembrane fluid flow component as a Dirichlet boundary condition. This approach allows for the control of fluid flow through deformable soft membranes without impeding other flow components.

We conducted creep and stress relaxation analyses on a numerical model of cylindrical hydrated soft tissue to validate the proposed approach. The results showed good agreement with the theoretical solutions derived by Soltz and Ateshian [21] (Figure 5). This indicates that the proposed approach can reproduce viscoelastic behaviors owing to the interaction between the interstitial fluid and solid skeleton of soft biological tissues, even for an elastic solid skeleton.

In addition, we performed indentation analyses on cylindrical hydrated soft tissue models enclosed by an impermeable membrane and found that the tissue volume remained constant during indentation, regardless of the amount of water enclosed in the tissue. This suggests that our new method, which successfully controls the fluid flow through deformable soft membranes and prevents the fluid from leaking out, can capture the conservation of mass.

### 4.2. Pitting Edema Indentation Test

Using a cylindrical analytical model, we replicated a pitting edema indentation test in a lower leg and analyzed the depth and duration of the pitting to assess the proposed method. By comprehensively integrating both fluid and solid behaviors over time and space, we observed the distinct behavior of pitting edema, characterized by a prolonged recovery time from compression [4], in the edematous state (Figure 6).

The pore water pressure was higher in the Edema state than that in the Normal state, supporting the skeleton and requiring a larger load for the same indentation depth as that in the Normal state. The expelled fluid gradually slowed as it extended from the loaded area, eventually halting at the top or bottom of the cylinder (Figure 7 and Figure 8). Subsequently, fluid accumulated at both ends of the cylinder, where it was restricted from passing through the boundary, resulting in an increased fluid volume fraction and mixture volume. The mixture primarily expanded radially, owing to the fixed length of the cylinder.

### 4.3. Factors Influencing Recovery Time in Edematous Tissues

The precise mechanism underlying the prolonged recovery time from indentation in edematous states remains unclear. It is speculated that tissue swelling caused by excess fluid expands voids within the tissue, potentially decreasing viscosity while increasing Darcy permeability. However, a decrease in viscosity would typically lead to faster recovery from indentation, rather than slower recovery. The deformation-dependent nature of the elasticity of the tissue skeleton may also contribute to faster recovery in the edematous state, given that the elasticities of biological soft tissues generally strengthen with greater strain [22].

To explore the factors prolonging recovery time in the edematous state, we conducted analyses assuming that the Darcy permeability of the tissue remains constant regardless of deformation, although the dependencies of permeability on deformation have been modeled in previous studies e.g., [13,14,23]. Subsequently, as mentioned previously, we successfully reproduced an extended recovery time from compression in the edematous state (see Figure 6). As depicted by the arrows in Figure 7 and Figure 8, fluid permeated throughout the tissue in both the Normal and Edema states. This observation suggests that tissue swelling expands the range of fluid movement. Consequently, in the Edema state, fluid must travel a greater distance to revert to its original state after unloading. This could explain the slower recovery from indentation observed in the Edema state. These findings were obtained as a result of our newly proposed method, which achieved the enclosure of fluid in deformable soft membranes without obstructing the flow parallel to the membrane. This method allowed the trajectory of the fluid, which permeated throughout the tissue, accumulated in distant regions, and returned after unloading, to be accurately tracked.

It is also plausible that the viscosity of the tissue skeleton becomes stronger under large deformation, thereby prolonging the recovery time. However, to the best of our knowledge, experimental results on the viscosity of the tissue skeleton have not yet been published. The viscosity of the tissue skeleton can be inferred from the fluid enclosed in the cells of the skeleton. In this case, the proposed method could also contribute to investigating the viscosity of the tissue skeleton.

### 4.4. Future Research

In our indentation analysis, we observed intricate phenomena in detail and proposed a candidate mechanism where excess fluid in tissues prolongs recovery time from compression. However, owing to the complexities of edema, flow dynamics under compressive loads are challenging to measure and visualize experimentally, resulting in a lack of experimental data for further verification. Moreover, to the best of our knowledge, detailed experimental results that could serve as sources of information on edema have not yet been published. While future experimental studies using ultrasonic and bio-impedance methods are planned, our proposed method offers an effective means to comprehend the edema response and aid in establishing feasible experimental methods. Understanding the internal lymphatic and blood fluid flow, pore pressure, and deformation in edematous conditions can enable the accurate assessment of lymphatic and blood stasis and flow disturbances, which are the primary causes of edema, thereby leading to the formulation of more effective prevention and treatment plans. In addition, changes in lymphatic and blood flow and pressure can serve as early indicators of edema to facilitate early symptom detection. Observing changes in the internal flow and pressure can also allow for an objective evaluation of the effects of new treatments or medications. Finally, obtaining information on the internal flow, pressure, and subtle deformations of lymphatic and blood fluids can optimize clinical planning for edemas requiring surgical intervention.

The current analysis demonstrates that our novel method can be useful for analyzing edemas. However, using models that accurately represent the anatomical positions and material constants of components such as the bones, fat, and muscles of the lower leg is necessary to achieve more quantitatively accurate analyses. To this end, we plan to implement larger-scale computations in the future.

## Figures and Tables

**Table 1:** Conditions of Normal and Edema states.

	Normal	Edema
Total volume (L)	0.785	1.02
Solid volume (L)	0.393	0.393
Fluid volume (L)	0.393	0.628
Initial pore water pressure (kPa)	0.00	4.50
Maximum load (kPa)	5.95	8.50

**Figure A1:**
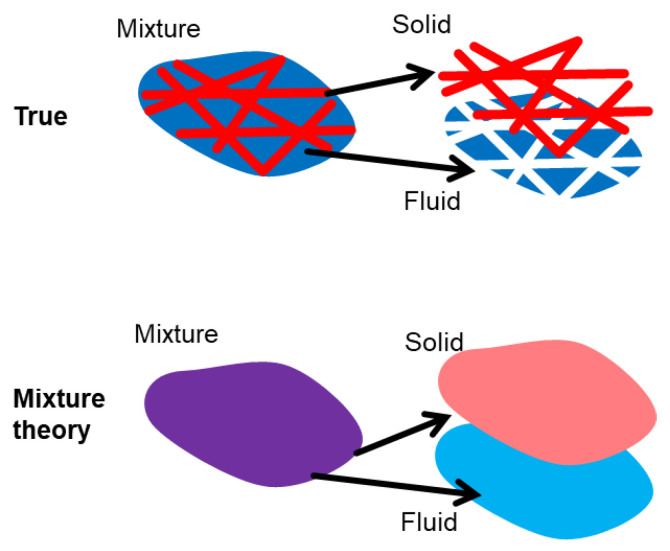
Apparent configurations of the fluid and solid phases.

## Data Availability

The datasets generated during the current study are available from the corresponding author on reasonable request.

## Appendix A. Governing Equations

### Appendix A.1. Piola Transformation

In mixture theory, a solid and fluid are assumed to be macroscopically continuous and to occupy the entire mixture space. Therefore, the fluid and solid phases in this study were assumed to occupy the same physical space simultaneously (Figure A1). As the fluid and solid phases move separately, the deformation gradient F and its determinant J can be defined using the undeformed solid phase as the reference configuration. Subsequently, the Piola transformation can be defined as follows:

(A1) $$A=JF^{-1}\cdot a,$$

where a denotes an arbitrary tensor. The relationship between the divergence of the Piola transformation and arbitrary tensor can be expressed as

(A2) $$\nabla_{\chi }\cdot A=J\nabla_{x}\cdot a,$$

where $\nabla_{\chi }$ and $\nabla_{x}$ represent the gradient operators with respect to the reference and current configurations, respectively [5].

### Appendix A.2. Momentum Equation

Assuming a quasi-static state, the body and inertial forces were neglected. The momentum equation of the mixture can be expressed as

(A3) $$\nabla_{x}\cdot \sigma =0,$$

where σ denotes the Cauchy stress tensor. As the mixture was assumed to be completely saturated with incompressible constituents,

(A4) $$\sigma =-pI+\sigma^{E},$$

where p denotes the pore water pressure, I indicates the rank-two identity tensor, and $\sigma^{E}$ represents the stress induced by the deformation of the solid skeleton, which is the drained solid constituent [10,13,19,24]. Using the first Piola–Kirchhoff stress Π, which is obtained from the Cauchy stress tensor σ based on the Piola transformation, Equation (A3) can be transformed into

(A5) $$\nabla_{\chi }\cdot \Pi =0;$$

(A6) $$\Pi =JF^{-1}\cdot \sigma .$$

Following manipulation using a virtual value of the solid phase displacement (δu) and the corresponding virtual value (δE), an integral formulation of Equation (A5) over a reference volume $\Omega_{0}$ of the mixture can be obtained, as follows [5]:

(A7) $$\int_{\Omega_{0}}^{\text{​}}S:\delta Ed\Omega_{0}=\int_{\Gamma_{0}^{t}}^{\text{​}}\left(\Pi^{t}\cdot N\right)\cdot \delta ud\Gamma_{0},$$

where $\Pi^{t}$ and N denote the prescribed surface traction and outward unit normal vector on the reference surface $\Gamma_{0}^{t}$, respectively. The second Piola–Kirchhoff stress S can be expressed as

(A8) $$S=JF^{-1}\cdot \sigma \cdot F^{-T}=-pJC^{-1}+JF^{-1}\cdot \sigma^{E}\cdot F^{-T}\equiv -pJC^{-1}+S^{E},$$

where $S^{E}$ represents the second Piola–Kirchhoff stress induced by the deformation of the solid skeleton.

### Appendix A.3. Continuity Equation

Assuming that both the solid and fluid phases are incompressible, the following equation was obtained [5]:

(A9) $$\nabla_{x}\cdot \left(v^{s}+w\right)=0,$$

where w denotes the fluid relative velocity that is defined as

(A10) $$w=\phi^{f}\left(v^{f}-v^{s}\right),$$

in which $\phi^{f}$ indicates the true volume fraction of the fluid, and $v^{f}$ and $v^{s}$ represent the velocity vectors of the fluid and solid, respectively. Using the Piola transformation, this equation can be transformed into

(A11) $$J\nabla_{x}\cdot v^{s}+\nabla_{\chi }\cdot W=\dot{J}+\nabla_{\chi }\cdot W=0,$$

where

(A12) $$W=JF^{-1}\cdot w.$$

Integrating this equation yields

(A13) $$J-1+\nabla_{\chi }\cdot Q=0,$$

where Q denotes the accumulated outflow volume vector of the fluid phase in the reference configuration, as follows:

(A14) $$W=\dot{Q}.$$

Introducing a virtual value (δp) yields an integral formulation of Equation (A13) over a reference volume $\Omega_{0}$ of the mixture, as follows:

(A15) $$\int_{\Omega_{0}}^{\text{​}}\delta p\left(J-1+\nabla_{\chi }\cdot Q\right)d\Omega_{0}=0.$$

### Appendix A.4. Darcy’s Law

We assumed that the motion of the fluid inside the mixture follows Darcy’s law with the Darcy permeability tensor of the mixture, κ:

(A16) $$w=-\kappa \cdot \nabla_{x}p.$$

As

(A17) $$F^{T}\cdot \nabla_{x}p=\frac{\partial x_{j}}{\partial \chi_{i}}e_{i}⊗e_{j}\cdot \frac{\partial p}{\partial x_{k}}e_{k}=\frac{\partial p}{\partial \chi_{i}}e_{i}=\nabla_{\chi }p,$$

where the Einstein summation convention is used, Equation (A16) can be transformed into

(A18) $$W=-Κ\cdot \nabla_{\chi }p,$$

where

(A19) $$Κ=JF^{-1}\cdot \kappa \cdot F^{-T}.$$

By introducing a virtual value (δQ), an integral formulation of Equation (A18) over a reference volume $\Omega_{0}$ of the mixture can be obtained as follows:

(A20) $$\int_{\Omega_{0}}^{\text{​}}\left[-\delta Q\cdot {Κ}^{-1}\cdot W-\delta Q\cdot \nabla_{\chi }p\right]d\Omega_{0}=\int_{\Omega_{0}}^{\text{​}}\left[-\delta Q\cdot {Κ}^{-1}\cdot W-\left\{\nabla_{\chi }\cdot \left(\delta Qp\right)-p\nabla_{\chi }\cdot \delta Q\right\}\right]d\Omega_{0}=0.$$

The following equation was obtained using the Gauss theorem:

(A21) $$\int_{\Omega_{0}}^{\text{​}}\left[-\delta Q\cdot {Κ}^{-1}\cdot W+p\nabla_{\chi }\cdot \delta Q\right]d\Omega_{0}=\int_{\Gamma_{0}^{p}}^{\text{​}}\delta Q\cdot \bar{p}Nd\Gamma_{0},$$

where $\bar{p}$ denotes the prescribed surface pressure on the reference surface $\Gamma_{0}^{p}$.

## Appendix B. Solid Volume Fraction

As the solid was incompressible and the undeformed solid phase was used as the reference configuration, the solid volume contained in the mixture was conserved. Therefore, the solid volume fraction $\phi^{s}$ can be obtained as

(A22) $$\phi^{s}=\phi_{0}^{s}/J,$$

where $\phi_{0}^{s}$ and J denote the initial solid volume fraction and volume change of the mixture, respectively.
